# Effect of Conditioning on PU Foam Matrix Materials Properties

**DOI:** 10.3390/ma15010195

**Published:** 2021-12-28

**Authors:** Lubomír Lapčík, Martin Vašina, Barbora Lapčíková, Yousef Murtaja

**Affiliations:** 1 Faculty of Technology, Tomas Bata University in Zlin, Nam. TGM 275, 760 01 Zlin, Czech Republic; lapcikova@utb.cz; 2 Department of Physical Chemistry, Faculty of Science, Palacky University Olomouc, 17. Listopadu 12, 771 46 Olomouc, Czech Republic; yousef.murtaja01@upol.cz; 3 Department of Hydromechanics and Hydraulic Equipment, Faculty of Mechanical Engineering, VSB-Technical University of Ostrava, 17. listopadu 15/2172, Poruba, 708 33 Ostrava, Czech Republic

**Keywords:** thermal analysis, polyurethanes, open-cell foams, mechanical vibrations, tensile testing, compression testing

## Abstract

This article deals with the characterization of the thermal-induced aging of soft polyurethane (PU) foams. There are studied thermal and mechanical properties by means of thermal analysis, tensile, compression and dynamic mechanical vibration testing. It was found in this study, that the increasing relative humidity of the surrounding atmosphere leads to the initiation of the degradation processes. This is reflected in the observed decreased mechanical stiffness. It is attributed to the plasticization of the PU foams wall material. It is in agreement with the observed increase of the permanent deformation accompanied simultaneously with the decrease of Young’s modulus of elasticity. The latter phenomenon is studied by the novel non-destructive forced oscillations vibration-damping testing, which is confirmed by observed lower mechanical stiffness thus indicating the loss of the elasticity induced by samples conditioning. In parallel, observed decreasing of the matrix hardness is confirming the loss of elastic mechanical performance as well. The effect of conditioning leads to the significant loss of the PU foam’s thermal stability.

## 1. Introduction

Polyurethanes are a group of polymers formed by the reaction of multifunctional isocyanates with polyalcohols. Polyurethanes (polycarbamates, or PU) are characterized by the (-NH-CO-O-) group [1]. In constitution and properties, they lie between polyureas (-NH-CO-NH-) and polycarbonates (-O-CO-O-). Polyurethane products are very different in nature. The diversity is attributable to the urethane bond’s qualities (-NH-C-O-O) and the existence of other groups in the chain, which enable the application of intermolecular forces, cross-linking, and crystallization orientation, flexibility, and chain stiffening [2]. While PU can be synthesized in various methods, it is always based on diisocyanates in practice. Due to the toxicity of isocyanate raw materials and their synthesis method utilizing phosgene, new synthetic routes for polyurethanes without using isocyanates have been developed at present [2].

The study of polymer thermal aging and degradation at specific humidity conditions reached a relatively high importance in technological praxis due to the wide applications of porous polyurethane based materials in automotive, aerospace [3], food packaging [4,5], and consumer products industries [6,7]. In general, polymers are thermally stable until reaching the decomposition threshold [8]. Two main types of polymer thermal decomposition processes are usually recognized for polymer main macromolecular chain: depolymerization and random decomposition. These two processes can occur concurrently or independently. On the other hand, individual polymers are heated in accordance with the specified time-temperature regimes and often experience melting first, followed by various stages of the thermal degradation process [8]. Temperature changes can stimulate a variety of physical and chemical processes in polymer systems. Important examples of these processes include thermal degradation, cross-linking, crystallization, glass transition, hydrolysis [9] etc.

Authors [10] found that the polyurethane biodegradation is controlled by diffusion of oxygen into the polymer. In PUs, the urethane or carbamate linkage is also susceptible to hydrolysis, as it is an ester linkage of a substituted carbamic acid. However, hydrolytic degradation occurs less readily than with the carboxylic ester linkage. The ultimate strain of the elastomers was decreasing with increasing degradation time.

Changes of the PUs fluorescence mapping signals induced by the weathering indicated degradation process proceeding in the PU matrix, as reflected in the increase in fluorescence intensity over time in the mapped fluorescence signals excited at 470 nm and detected in a wavelength range exceeding 500 nm. These signals revealed as response to the ongoing progress of polymer ageing [11].

Most polyurethanes are derived from three basic building blocks: a polyol (a multifunctional alcohol, usually polymeric, e.g., polybutadiene with hydroxyl-terminated hydroxyls), a diisocyanate, a chain extender (e.g., low molecular weight or polymeric diol or diamine), and a cross-linker (e.g., triol). Two basic procedures are used to synthesize polyurethanes: one-step and two-step, i.e., either a one-step mixing of all components of the reaction mixture or a prepolymer preparation procedure. One-step synthesis of polyurethanes is carried out by simultaneous mixing of polyol, diisocyanate (MDI, TDI), extenders (diols, diamines), and cross-linkers (multifunctional alcohols or amines) [12]. Cross-linking is often carried out with sufficient speed at room temperature and is completed within about a few hours. The reaction time can be shortened as required by adding accelerators (dibutyltindilaurate, stannous octoate, etc.), which are added just before application. The two-step synthesis produces the final polyurethane in two temporally separated reaction steps. In the first stage, the polyol reacts with the diisocyanate to form an intermediate with NCO-terminal groups, the so-called prepolymer. In the second step, the prepolymer reacts with extenders and cross-linkers to form the final product. In general, the prepolymer can be prepared with any excess isocyanate (isocyanate index rNCO/OH > 2). The viscosity of the prepolymer increases strongly (up to 5 times for the original hydroxyl-terminated polybutadiene); the excess liquid isocyanate can prevent or mitigate this increase (dilution effect). The hydroxyl-terminated polybutadiene may be partially replaced by a proportion of polyester diol, polyether diol, or polyol in general. From a homogeneity standpoint, it is frequently preferable to convert these components of the mixture independently into prepolymers before combining. The two-step method improves the characteristics of crosslinked matrices [6].

Presently, the research and development of recyclable and sustainable PU foams are focused on advanced bio-based systems in packaging application, e.g., for refrigerated and frozen foodstuff storage and sale. For this reason, the modern trend in the field is moving towards fully bio-based and compostable foams. However, the direction via partially bio-based PU foams remains the most consistent performer [13]. For example, the application of the algae and algal cellulose added during PU-composite foam production led to the algae base cellulose integration into the PU foam structure and resulted in more open cells and softer foam structure. The PU-composite showed greater shock absorbent capacity in comparison with the usual PU foam suitable for the application in the food packaging industry [14]. Another current trend in PU material development is the non-isocyanate PU foam synthesis [15,16].

According to the structure, soft and rigid PU foams with different specific gravity, porous structure, and mechanical properties are distinguished. The basic components of soft foams are a mixture of 2,4- and 2,6-toluenedioisocyanate and polyols (polyesters-linear or slightly branched, polyethers). The main blowing agent is gaseous carbon dioxide, formed by the reaction of water with the isocyanate group. This reaction produces highly polar groups, forming H-bridges and affecting physical properties. Soft PU foams have a foam structure with open, interconnected pores. Rigid PU foams are networks with denser interchain bonds or cross-linking sites, and a higher content of rigid urethane sections. The closed pore structure predominates in the foam because the pore walls are more rigid. The mechanical strength of the foam increases with specific gravity. Soft PU rubbers, cross-linked elastomers and soft PU foams have approximately the same chain stiffness. High chain stiffness and intermolecular forces characterize rigid PU foams.

Results of the vibrational dynamical mechanical analysis combined with the FTIR analysis allowed detailed understanding of the induced chemical changes leading to both the crosslinking, as well as degradation processes, in the complex PU matrix [17,18]. The final changes of the mechanical properties were dependent on the type of PU matrix, where poly(ester) based PUs exhibited higher stiffness in comparison with the poly(ether) [19].

The combination of the novel, non-destructive dynamic-mechanical vibrational analysis with the conventional tensile, hardness, and permanent deformation measurements was applied in this study. The latter experimental techniques were combined with the thermal analysis. Studied samples were subjected to varying conditioning times at different temperatures and relative humidity.

## 2. Materials and Methods

### 2.1. Materials

**Figure 1:**
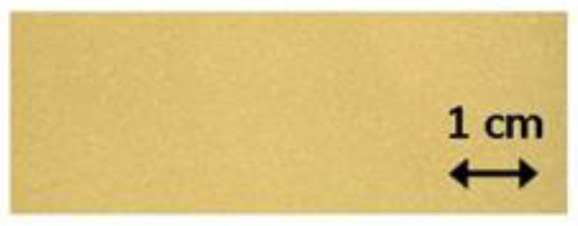
Photo of the studied PU foam material.

Open cell soft PU foams of (35.4 ± 0.3) g/cm^3^ density were purchased in the local construction hobby market and studied. PU samples were cut into the required shapes and dimensions of the specimen, e.g., dog bone for tensile, cubic for mechanical vibration analysis, permanent deformation, and hardness testing. A photo of the studied foam is shown in Figure 1. Small bits of the tested PU foams were used for thermal analysis. All materials under study were subjected to the conditioning at two different temperatures of 45 and 80 °C and relative humidity kept at 45 and 80% for different time intervals ranging from 0 to 300 h in the climate chamber Discovery 105 (Angelantoni Test Technologies, Massa Martana, Italy).

### 2.2. Methods

Uniaxial tensile testing was performed on Autograph AGS-X instrument (Shimadzu, Kyoto, Japan) equipped with the Compact Thermostatic Chamber TCE Series. Measurements were performed according to the ČSN EN ISO 527-1, 527-2 standards at 100 mm/min deformation rate [20]. Each measurement was repeated ten times and mean values were calculated.

Thermogravimetry (TG) and differential thermal analysis (DTA) experiments were measured on simultaneous DSC/TGA, SDT 650 Discovery with TRIOS software for thermal analysis (TA Instrument, New Castle, DE, USA). The apparatus was calibrated using indium as a standard. Prior to each measurement, samples were grated into an aluminum pan. Throughout the experiment, the sample temperature and weight-heat flow changes were continuously monitored. The measurements were performed at a heat flow rate of 10 °C/min in a static air atmosphere at the temperature range of 30 to 300 °C.

The material’s ability to damp harmonically excited mechanical vibration of single-degree-of-freedom (SDOF) systems is characterized by the displacement transmissibility *T_d_*, which is expressed by the Equation (1) [21]:

(1) $$T_{d}=\frac{y_{2}}{y_{1}}=\frac{a_{2}}{a_{1}}=\sqrt{\frac{k^{2}+{\left(c\cdot \omega \right)}^{2}}{{\left(k-m\cdot \omega^{2}\right)}^{2}+{\left(c\cdot \omega \right)}^{2}}}$$

where *y*_1_ is the displacement amplitude on the input side of the tested sample, *y*_2_ is the displacement amplitude on the output side of the tested sample, *a*_1_ is the acceleration amplitude on the input side of the tested sample, *a*_2_ is the acceleration amplitude on the output side of the tested sample, *k* is the stiffness, *c* is the damping coefficient, *m* is the mass, and *ω* is the angular frequency [22,23].

The mechanical vibration damping testing of the investigated PU foams was performed by the forced oscillation method [24]. The displacement transmissibility *T_d_* was experimentally measured using the BK 4810 vibrator combined with a BK 3560-B-030 signal pulse multi-analyzer, and a BK 2706 power amplifier at the frequency range from 2 to 1000 Hz [25]. The acceleration amplitudes *a*_1_ and *a*_2_ on the input and output sides of the investigated specimens were evaluated by means of BK 4393 accelerometers (Brüel & Kjær, Nærum, Denmark) [26]. The tested specimen dimensions were (60 × 60 × 50) mm (length × width × thickness). Each measurement was repeated three times at an ambient temperature of 25 °C and mean values of the displacement transmissibility were calculated.

Foam hardness was measured according to ČSN EN ISO 2439 (645440) standard method A “Flexible cellular polymeric materials-Determination of hardness (indentation technique)”. During the hardness measurements, the specimens were compressed to 60% of their original height, and the force value (N) was read after 30 s of the deformation. The tested specimen dimensions were (50 × 50 × 50) mm (length × width × thickness). Each measurement was repeated five times at selected conditioning temperatures of 45 and 80 °C and relative humidity of 40 and 80%.

Permanent deformation was measured according to ČSN EN ISO 1856 standard. The tested block specimen dimensions were (50 × 50 × 50) mm (length × width × thickness). Each measurement was repeated five times. Tested samples after conditioning for 300 h at the same temperatures and relative humidity as given in the section above were inserted between the plates of the pressing device compressed to 50% of the original dimensions for 30 min. At the end of the period of compression time, the specimens were released and left lying freely on the table for 30 min. The thickness was then measured again and the permanent deformation expressed as a percentage relative to the original samples dimension was calculated.

## 3. Results

**Figure 2:**
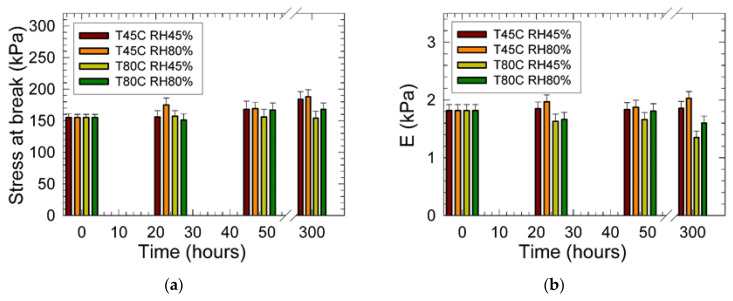
Results of the uniaxial tensile testing of the studied PU materials: (**a**) Stress at break vs. conditioning time at different temperatures and relative humidity; (**b**) Young´s modulus of elasticity (*E*) vs. conditioning time at different temperatures and relative humidity. Inset: T—temperature (°C), RH—relative humidity (%).

Results of the tensile testing of the studied PU foam materials are shown in Figure 2. Observed data indicated an increase in mechanical strength and plasticity of the cellular materials after 300 h conditioning as reflected in the increased magnitudes of the stress at break by 19% from 155 kPa to 184 kPa (for sample treated at 45 °C and 80% relative humidity). Simultaneously, the increase in ductility from 87% (at the conditioning temperature of 45 °C and the relative humidity of 45%) to 114% (at the conditioning temperature of 80 °C and the relative humidity of 45%) was observed. For the conditioning temperature of 80 °C, this dependence remained relatively constant. For the relative humidity of 80% and the conditioning temperature of 45 °C, the mechanical strength after 24 h increased, followed by the sharp decline observed after 48 h of conditioning. However, it increased again after 300 h of conditioning. Latter mentioned behavior indicated that at 80% relative humidity, significant structural changes ascribed to the cross-linking were obtained after 48 h of conditioning. At the temperature of 80 °C and a relative humidity of 45%, the stress at break did not change significantly. However, it increased about 10% at the higher relative humidity conditions.

Figure 2 shows that the PU matrix continued to plasticize, as seen by the rise in ductility of the material. We assume that this may be a combination of a change in the elasticity of the individual cell walls of the foam matrix. However, we also have to consider the resulting air pressure increase induced by the change in individual cell volumes during the deformation of the foam structure. The latter change of volume is also associated with a change in the geometry of the individual cells. Simultaneously, the change in the viscoelastic and viscoplastic properties of the PU matrix should be assumed due to the PU matrix cross-linking [27], and partial hydrolysis [28]. As a complex material response, the varying degrees of swelling of the PU cell walls material were proposed. We assume that the viscous friction and the wall stiffness are the dominant mechanisms of the transferred mechanical energy dissipation [29].

The observed increase of the stress at the break for high relative humidity conditioning (80%) at 45 °C indicated an increase of the PU matrix’s stiffness, accompanied by the increase of Young´s modulus of elasticity resulting in a simultaneous increase in matrix brittleness. However, signs of matrix hydrolysis were identified at 80 °C conditioning for both relative humidity conditions resulting in the decrease of the *E* from 1816 Pa (original sample without conditioning) to 1350 Pa (conditioned at 80 °C and 45% RH) and to 1600 Pa (conditioned at 80 °C and 80% RH).

**Figure 3:**
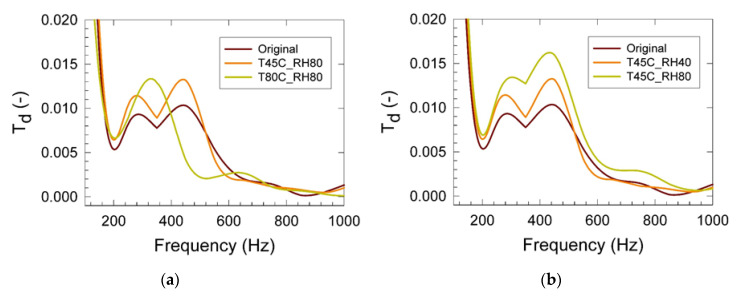
Displacement transmissibility vs. frequency as obtained from the vibration damping measurements of the studied PU materials after 300 h conditioning: (**a**) Effect of the conditioning temperature at 80% RH; (**b**) Effect of the conditioning relative humidity at 45 °C. Inset: T—temperature (°C), RH—relative humidity (%), Original—non-conditioned sample.

**Figure 4:**
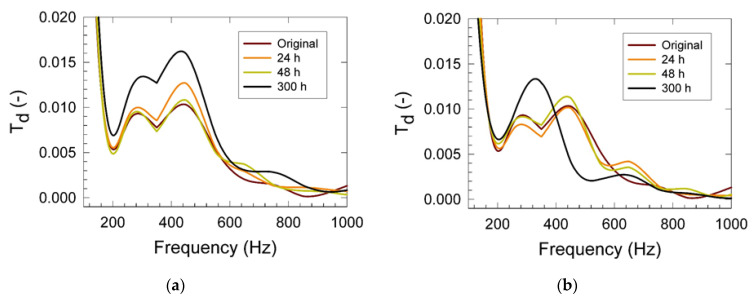
Displacement transmissibility vs. frequency as obtained from the vibration damping measurements of the studied PU materials: (**a**) Effect of the conditioning time at 45 °C and 80% RH; (**b**) Effect of the conditioning time at 80 °C and 80% RH. Inset: Conditioning time (hours), Original—non-conditioned sample.

Results of the mechanical vibration measurements are summarized in Figure 2 and Figure 3. It was found that the displacement transmissibility gradually increased with the increasing conditioning temperature and high relative humidity of 80% (Figure 3a), indicating higher mechanical stiffness of the PU matrix in the frequency range of 250 to 400 Hz. A similar effect was also found for the 40% relative humidity conditioning. We assume that the chemical cross-linking process of the residual non-reacted PU matrix components proceeded in the time scale of the first 48 h of the conditioning treatment. This hypothesis was also supported by our previous tensile testing measurements discussed above. Simultaneously, the increasing trend of the PU matrix mechanical stiffness was also observed. This was reflected in the increased magnitude of *T_d_* with the increasing relative humidity, as shown in Figure 3b [30]. Results shown in Figure 4a indicated increased elasticity with the increased conditioning time of the studied foams as reflected in the appearance of the two maxima of *T_d_* (at the frequencies of 280 and 430 Hz and magnitudes of 0.014 and 0.017). Observed stiffening appeared in the relatively wide frequency range of 200 to 600 Hz. After prolonged conditioning, plasticization patterns in mechanical behavior were found, as indicated in Figure 4b. After 24 h of conditioning at 80 °C and 80% RH, the foam elasticity was decreased in the frequency range of 200 to 600 Hz in comparison with the virgin PU sample indicating the probable start of the hydrolysis process of the wall material decomposition [31,32].

**Figure 5:**
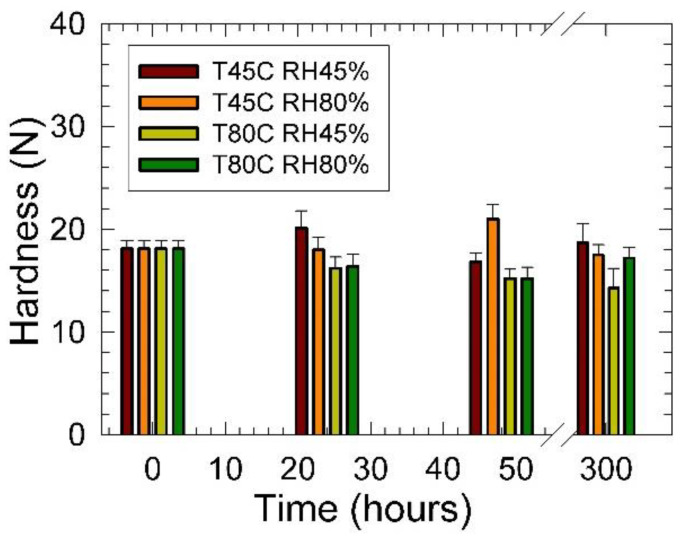
Hardness vs. conditioning time dependencies of studied PU foams at different temperatures and relative humidity. Inset: T—temperature (°C), RH—relative humidity (%).

Results of the effect of the sample conditioning on PU foam hardness are shown in Figure 5. It was found that with increasing conditioning time, the hardness decreased for all tested materials except conditioning at 45 °C. Most probably, this fact was attributed to the increased PU foams wall matrix crosslinking density as obtained at latter mentioned conditioning parameters. After 300 h conditioning in the temperature range of 45 to 80 °C and at the relative humidity of 45 and 80%, the hardness decreased from 10 to 18%. However, at the 45 °C temperature and the relative humidity of 45%, the hardness slightly increased from 18 N to 19 N. We assumed the onset of the PU molecules degradation due to increased temperature and relative humidity. It was found by Weise et al. [19] that crosslinks occur to a greater extent for the PUs containing more poly(ester) because the ester groups can more easily undergo hydrolysis than the PUs containing more poly(ether). It was confirmed by FTIR analysis [19], that the hydrolysis of ester groups yields carboxylic acids, which provide new and rigid ionic crosslinks. In the case of PUs containing only the ether macrodiols, they became slightly more mechanically stiff at the short weathering time less than 1000 h followed by their weakening to the point of fracture after prolonged weathering. This indicated that although shorter and more ridged crosslinks were formed during weathering, the sum of the degradation ultimately weakened the mechanical properties of poly(ether) type PUs [19].

**Figure 6:**
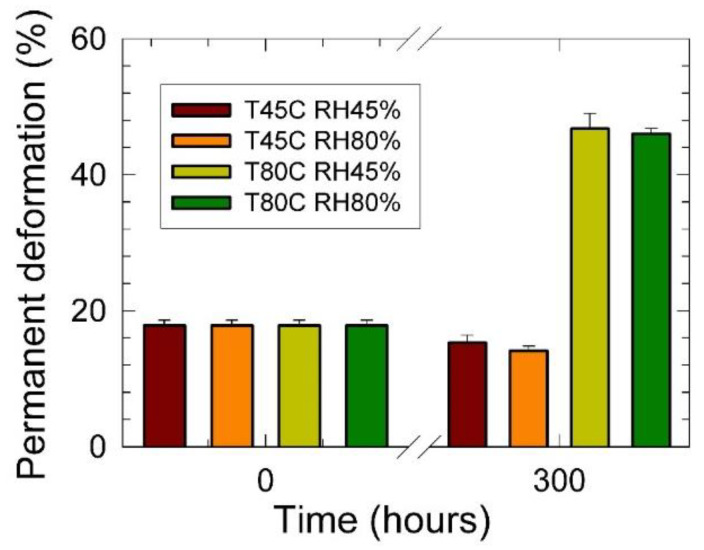
Permanent deformation vs. conditioning time dependencies of studied PU foams at different temperatures and relative humidity. Inset: T—temperature (°C), RH—relative humidity (%).

Results of the permanent deformation measurements are shown in Figure 6. It was found that conditioning at the lower temperature of 45 °C triggered higher elasticity of the PU foams independently of the surrounding atmosphere relative humidity. These results were in excellent agreement with the observed increased Young´s modulus of elasticity, as shown in Figure 2b. With increased conditioning temperature, the permanent deformation vigorously increased from 18% (for the original sample) to 46% after 300 h of conditioning time. The latter permanent deformations were independent of the applied relative humidity. Again, these results agreed with the obtained decrease of the *E* reflecting loss of elasticity and increased plastic mechanical behavior.

It was found in the literature [19], that the single component PUs containing either a poly(ester) or poly(ether) macrodiols were mechanically more unstable than the two component PUs containing both macrodiols. This led to a synergistic effect where their damping abilities increased and their mechanical stabilities after weathering increased as well. The increase in the damping abilities of the blended PUs was attributed to a decrease in the packing efficiency of the macrodiols chains by combining the two different functional groups. Furthermore, the increase in the mechanical stabilities after weathering was observed as a result of the competing degradation processes and simultaneous protection of the urethane group by the ester.

**Figure 7:**
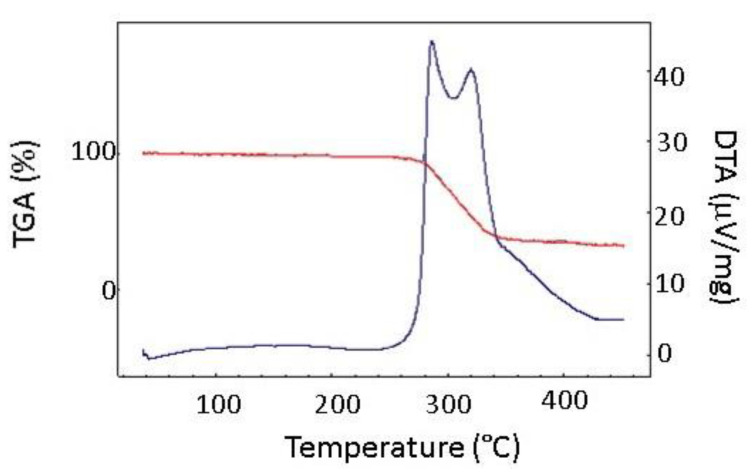
Typical TG DTA pattern of the studied PU foam material: red line—TGA, blue line—DTA.

A typical degradation pattern of the studied PU foams is shown in Figure 7. The degradation process was started at the temperature of 260 °C with the observed weight loss of about 86%, accompanied by the exothermic heat of fusion. Observed degradation pattern was ascribed to the decomposition of the PU material into the starting polyol and diisocyanate. It was followed by the thermal decomposition of the diisocyanate. Benzonitrile, methylbenzonitrile, phenyl isocyanate, toluene, and benzene were detected as the main decomposition products [33,34]. The latter decomposition process reflected the decomposition of diisocyanates and polyols in the PU matrix accompanied by the evolution of gaseous carbon oxide and hydrogen cyanide [7].

**Figure 8:**
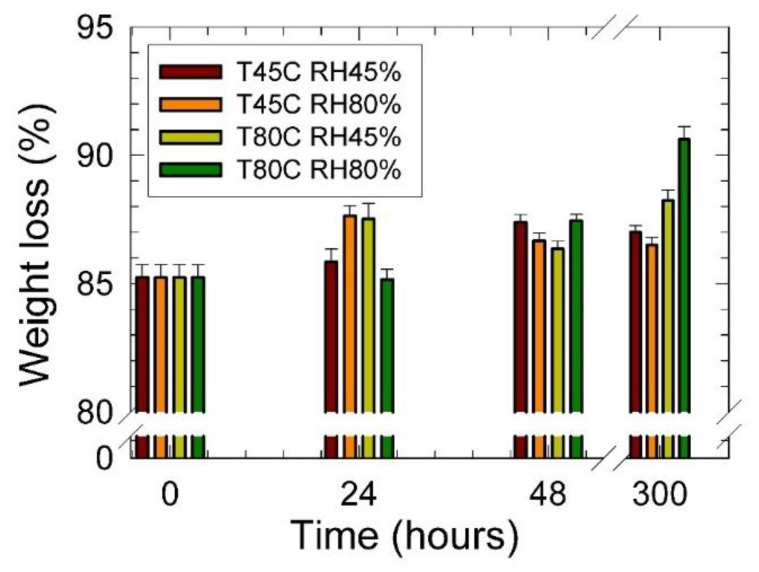
Weight loss vs. conditioning time of the studied PU foams. Inset: T—temperature (°C), RH—relative humidity (%).

In Figure 8 results of the TGA analysis are summarized. Observed data shows a minor increase in the weight loss with the increasing conditioning time for both conditioning temperatures and relative humidity. It was ascribed to the change of the chemical composition of the PU foam, most probably due to the addition of the free isocyanate groups. It can be concluded that conditioning led to the loss of the PU foam’s thermal stability.

## 4. Conclusions

This study was aimed on the physicochemical and mechanical characterization of the thermal-induced degradation of the commercial soft polyurethane foams. It was found that the increased conditioning temperature and the relative humidity of the surrounding atmosphere led to the initiation of the degradation patterns of the studied PU foams. There was an observed decrease in mechanical stiffness due to the plasticization of the PU foams wall material, as confirmed by the simultaneous increase of the permanent deformation accompanied by the decrease of the Young’s modulus of elasticity. This phenomenon was also confirmed by the nondestructive dynamical-mechanical vibration testing, which confirmed samples´ higher vibration damping, resulting in the loss of elasticity. The last changes of the mechanical behavior agreed with the observed decrease of the matrix hardness, again confirming the loss of elastic mechanical performance. It has been also found that the effect of conditioning led to the significant loss of the PU foam’s thermal stability.

## Data Availability

Not applicable.
